# A framework for test measurement selection in athlete physical preparation

**DOI:** 10.3389/fspor.2024.1406997

**Published:** 2024-07-01

**Authors:** Lachlan P. James, Jade A. Z. Haycraft, David L. Carey, Samuel J. Robertson

**Affiliations:** ^1^ Sport, Performance, and Nutrition Research Group, School of Allied Health, Human Services, & Sport, La Trobe University, Melbourne, VIC, Australia; ^2^ Institute for Health and Sport, Victoria University, Footscray, VIC, Australia

**Keywords:** sport, assessment, validity, determinants, strength and conditioning

## Abstract

Preparing athletes for competition requires the diagnosis and monitoring of relevant physical qualities (e.g., strength, power, speed, endurance characteristics). Decisions regarding test selection that attempt to measure these physical attributes are fundamental to the training process yet are complicated by the myriad of tests and measurements available. This article presents an evidenced based process to inform test measurement selection for the physical preparation of athletes. We describe a method for incorporating multiple layers of validity to link test measurement to competition outcome. This is followed by a framework by which to evaluate the suitability of test measurements based on contemporary validity theory that considers technical, decision-making, and organisational factors. Example applications of the framework are described to demonstrate its utility in different settings. The systems presented here will assist in distilling the range of measurements available into those most likely to have the greatest impact on competition performance.

## 1 Introduction

The physical preparation of athletes requires a determination and assessment of relevant physical qualities (e.g., strength, power, speed, endurance characteristics) to identify strengths or weakness, and inform training interventions (1). Decisions regarding test selection that seek to measure these physical qualities are therefore a critical part of the training process (2). This process is increasingly more complicated given the large volume of tests and outputted measures available to performance staff. As such, a systematic decision-making framework to select context-specific physical testing measures that guide athlete preparation for sport is needed for practitioners (e.g., strength and conditioning coaches, sport scientists, performance analysts, and applied researchers). Conceptualization of validity as a network of inferences (3) and through measurement, organizational and decision-making perspectives can provide guidelines to practitioners when navigating the measurement selection process (4). However, a solution has yet to be presented in the literature in the context of athlete assessment models. As such, this article will use a contemporary understanding of validity to build a framework to guide test measurement selection for the physical preparation of athletes, that considers technical, decision-making, and organizational factors.

## 2 Validity

Validity is a primary consideration in test measurement selection as it considers the degree to which a test or measure is capable of achieving specific aims. Traditionally, three forms of validity were recognized: content, criterion, and construct (5). However, in the past 50 years, numerous forms of validity have been described in the literature, with definitions changing over this time (6). This has evolved further into a unified theory which places less emphasis on delineating dozens of types of validity, in favor of the few original forms and the accumulation of evidence for and against a validity proposition and situational context (4). In the context of sports performance, the forms of validity used to determine the relevance of a particular test measurement to sport performance is dependent on the sport and performance context. In measured sports (i.e., timed, weight, distance), for which deterministic models can be generated (7), criterion validity may be readily applied and simply assessed to establish this link. For example, a measure of barbell velocity during a power clean at a fixed load may be able to provide an accurate indication of weightlifting performance during competition. However, most sports can be classified as non-deterministic or complex, where competition outcomes are a multifactorial construct of events (e.g., team, field, court, and combat sports) (8), as such, criterion validity of a physical performance metric may not be easily assessed. In non-measured sports, content validity can refer to the degree to which the measurements included in a performance testing battery quantify the intended physiological characteristics that are important for sport performance (9). However, a crucial step in the validation process is to determine the extent to which physical performance qualities contribute to sport performance outcomes. Further, an analysis is required to identify which of these qualities are most relevant in a given context. In these cases, construct validity is a key lens through which to view validity, where the goal is to establish the relevance of a physical quality to sport performance. To represent this complex process, construct validity of physical performance metrics may be represented as a series of inferences that link from a test score to the performance outcome of interest.

## 3 Layers of validity

**Figure 1:**
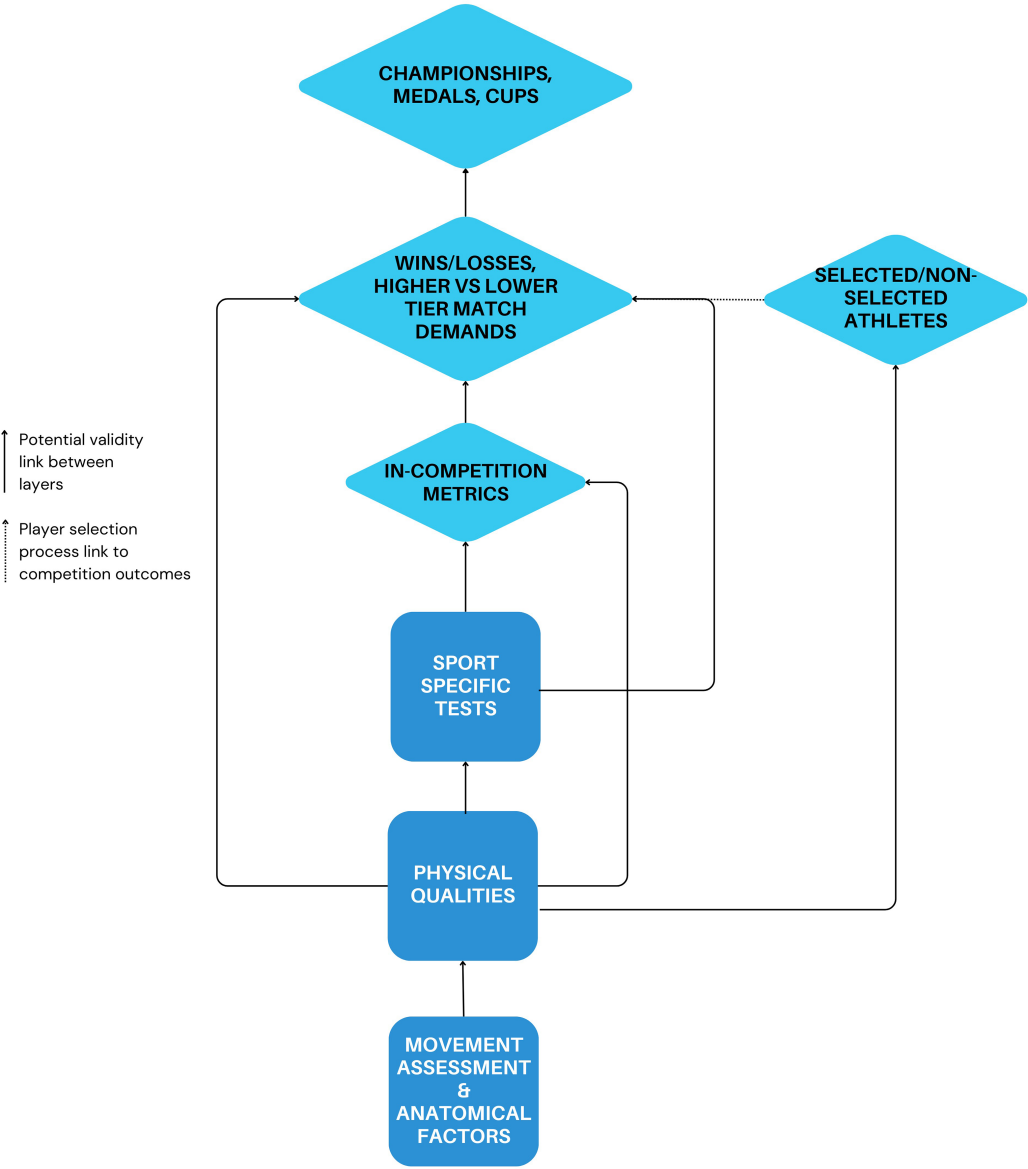
A multi-layered validity model, with each arrow representing a link of validity between performance measures.

**Figure 2:**
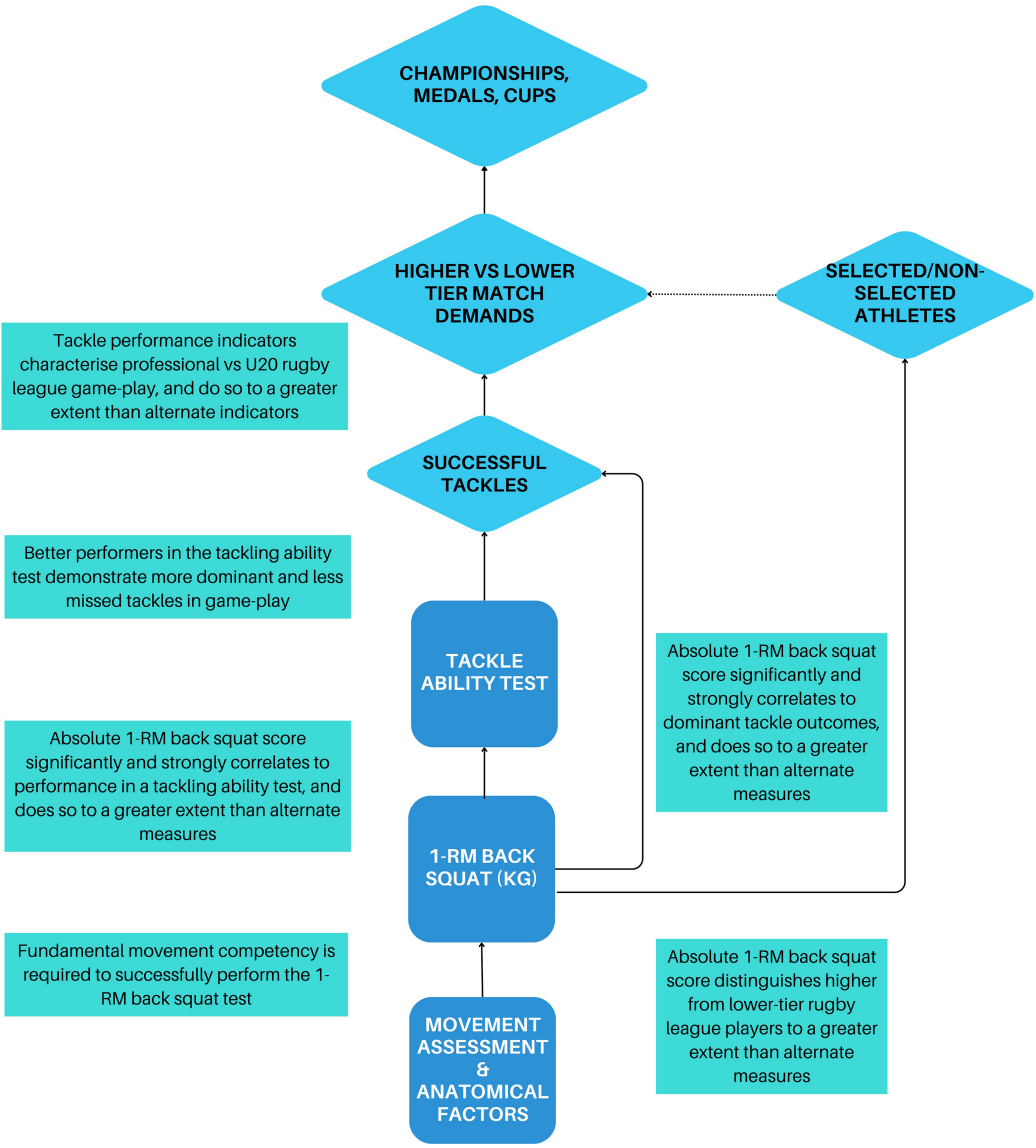
A case example of how multiple layers of evidence can be used to bridge the gap between the physical performance measurements and the construct of successful competition performance outcomes.

A network of inferences (3) or “layers of validity” can address the complexity of establishing measurement validity to the physical preparation process (Figure 1). This idea acknowledges that it may not be possible to establish a direct or causal relationship between a physical fitness measurement and successful competition performance outcomes (constructs) due to the complexity or abstract nature of sport competition. Instead, the relationship between physical performance measurements and competition outcomes may be assessed via intermediate steps or proxies that ultimately connect it to a competition performance outcome. For example, it may not be possible to determine whether a higher 1-RM back squat score leads to more wins in professional rugby league, but instead may be associated with within-game measures of tackling ability (10), which are in turn characteristic of higher- vs. lower-tier match-play (11). Furthermore, absolute 1-RM back performance distinguishes professional from semi-professional rugby league players to a greater extent than alternate metrics (12) (Figure 2). In this case, multiple layers of evidence are used to bridge the gap between the physical performance measurements and the construct of successful competition performance outcomes.

The process of establishing layers of validity involves breaking down the relationship between the physical performance measurements and the competition performance outcomes into a series of smaller, more manageable relationships or steps, each with its own validity evidence. By demonstrating the validity of these intermediate steps, the overall validity of the measure with respect to the intended construct can be supported indirectly. As the number of intermediate steps between the metric and the competition outcome increase so too does the complexity of the argument (13). However, this process allows for the accumulation of evidence towards the relationships between a measure and the sport performance outcome (14). Although a multi-layered validity model may differ somewhat based on the context, the objective is to provide a schematic of measurement-performance interaction that guides the analytical approach for constructing a validity argument. Therefore, it is also necessary to have a complementary framework to determine the *extent to which* one layer is connected to another.

## 4 Physical assessment framework

The objective of the framework is to evaluate the ability of a given test measurement to link the steps within the multi-layered validity model. The items listed within the framework are informed by contemporary validity theory (4) and therefore considers three primary criteria: (i) measurement (Table 1), (ii) decision-making (Table 2), and (iii) organizational (Table 3). The measurement criteria assess how a metric is collected, the quality and type of evidence supporting connections to performance outcomes, and the amount of unique information it captures (5). Decision-making is captured in the second criteria and considers the extent decisions can be made based upon the measurement (15). The organizational criteria accounts for the feasibility of implementing the test and measurement within the sport organization (16). The primary objectives of this framework are supplemented with secondary criteria that identify future side-effects or consequences (positive or negative) of utilizing the measure of interest that extend beyond the identified primary objectives (4). Also informing the framework are previous models developed to guide the implementation of sports technology (17, 18), and jump assessments (19), across contexts. Taken together, the proposed framework provides a systematic decision support process for sports practitioners that incorporates the technical and organizational needs of measurement selection for the physical preparation of athletes.

**Table 1:** Measurement criteria.

Measurement criteria	Definition	Scale
Metric collection	How valid and reliable is the proposed method of metric collection?	Low/Moderate/Strong
Evidence of association	How much, and what kind of evidence is there for a proposed link between a test metric and a performance outcome? Is there a plausible mechanistic explanation grounded in domain knowledge?	Low/Moderate/Strong
Nature of association	Has the type of association been explored?	Low/Moderate/Strong
Independence of information	Is the proposed metric measuring something already captured by another metric in the model?	No/Somewhat/Yes

**Table 2:** Decision making criteria.

Decision making criteria	Definition	Scale
Interpretability	Interpretability of physical performance metrics is the degree of meaningful insights that can be extracted from the metric, or a change in the metrics value can be used by coaches, sport science staff, and broader organization stakeholders to inform decision-making.	Low/Moderate/Strong
Responsiveness	The ability for a measurement to detect real changes to strength and conditioning interventions over time. Question that may be asked by the practitioner are: Can the measure inform training interventions? Is the measured attribute modifiable?	Low/Moderate/Strong
Diminishing returns	As a person receives more exposure to a given stimulus, their ability to adapt generally diminishes. Training may therefore be directed to an alternate, but still relevant physical quality. How well does the measure consider this criterion?	Low/Moderate/Strong

**Table 3:** Organizational criteria.

Organisational Criteria	Definition	Scale
Financial cost	The financial cost is as any monetary cost associated with physical performance measurements. This may include, but is not limited to, purchase and maintenance of equipment, venue cost and overheads (i.e., utility costs, internet), and staff wages for data collection, processing, analysis, and reporting. The financial costs of physical performance measures should remain within the budgetary constraints of the sports organization or governing bodies (i.e., soft capping of team finances, government sport funding)	Low cost, moderate cost, high cost
Opportunity cost	The opportunity cost of implementing a physical performance metric test are resources that may need to be sacrificed from other areas of sport program to allow a specific physical performance metric to be implemented. Opportunity costs may include the re-allocation of financial costs from other departments (i.e., nutrition, sport psychology, analytics), or sacrificing of time from training sessions (i.e., match-simulation, skills sessions, sport-specific training) to accommodate the physical performance testing session	Low cost, moderate cost, high cost
Time cost of test familiarization	The time cost of test familiarization considers the test complexity when implementing and administering a test. Increased task complexity within performance tests may require higher levels of expertise which incur a time cost in upskilling staff (i.e., equipment, software, pass/fail criteria) and athletes (i.e., pacing strategies, coordination of multi-joint complex movements) on test methods	Low cost, moderate cost, high cost
Time-cost of implementing in the training environment	Time-cost of test implementation considers timing and duration of physical performance testing within a training session, week, annual or bi-annual plan. Within the time-cost of test implementation, recovery time cost is also considered athletes to mitigate risk of injury or overtraining from residual fatigue between tests, testing days, and training days	Low cost, moderate cost, high cost

### 4.1 Measurement criteria

#### 4.1.1 Measurement collection

A metric must be viewed in the context of how it is captured, so the validity and reliability of the collection method is an essential consideration (20, 21). For example, linear position transducers have been shown to be a more valid and reliable method to measure barbell velocity during resistance training than accelerometer based devices (22). Metrics without evidence of reliability have limited utility for decision making within a validity model since variation may come from sources external to the athlete, such as measurement error, the identity of the person running the test, or the testing environment (23). Knowledge of the measurement error for a test is important to contextualize the size of any observed changes, and to avoid over-interpretation of individual changes that are small relative to the measurement error. Without validity it is difficult to understand the mechanisms of action between layers in the validity model and thus make targeted interventions. For example, an invalid test of change of direction ability may still show associations with performance outcomes if the majority of the variance is explained by a different physical property (such as speed) (24), however subsequent decision-making regarding training interventions may be targeted at the wrong physical qualities. This consideration has become acutely important with advances in technology and over-saturation in the market of measurement devices in sport (17).

#### 4.1.2 Evidence of association

Assessment of the strength of evidence supporting a connection between a test metric and performance outcome is multi-facetted and often challenging (25). A connection should ideally be supported by both a plausible mechanistic explanation and supporting experimental or generalizable observational data (26). Explanations grounded in domain knowledge of physiology and sport performance are important for establishing causal links and for interpreting published data (26, 27), and considerations of causality are necessary for the design and implementation of training interventions. For example, a study of recreational and untrained cyclists reported a strong correlation (*r* = 0.965) between maximum power in an incremental test (cycling to exhaustion from 100 W, increasing at 20 W·min^−1^) and functional threshold power (maximum average power sustained over a 1 h period) (28). Additionally, the study found that both the test value and functional threshold power significantly increased after a training intervention. In this example the practitioner can be confident that improvements in the incremental test will translate to performance gains in sub-elite cyclists. However, the generalizability of this finding to elite cohorts is not guaranteed since the trainability of the underlying physical qualities may be different, highlighting the importance of appraising evidence of association relative to the cohort considered. In addition to the presence of a mechanistic explanation, the body of empirical evidence supporting a link should be appraised within the traditional sources of evidence hierarchy (29), with attention given the magnitude of effects interpreted in the context of the desired outcome (30). Study population characteristics are another important consideration, and preference may be given to selecting metrics with associations shown within the same sport specialization and level of proficiency.

#### 4.1.3 Nature of association

After establishing an association, further understanding of how a metric is related to a performance outcome can inform the construction of the multi-layered validity model. Aspects to look for in the evidence include methods that allow for non-linear relationships, interacting (31), mediating (32, 33), or moderating (34) effects. Analytical techniques such as machine learning algorithms can permit for this non-linearity and complexity in metrics to be uncovered, however the use of such analysis methods does not supplant the need for plausible mechanistic and causal explanations (35). Examples include the identification of tipping points (e.g., the point between functional and non-functional overreaching), feedback loops (how an increase in maximal strength allows for enhanced adaptations to power training), or emergence (performance a live tester attacker vs. defender test can discriminate between higher and lower-level players, while a preplanned change of direction test cannot) (36). Having an awareness of these phenomena [see (36) for further reading on the topic], along with appropriate analytical tools to account for them can assist the strength and conditioning coach when selecting valid physical performance metrics within the team sport environment.

#### 4.1.4 Independence of information

In some cases, a set of multiple metrics may be assessed to have equal measurement validity, reliability, and association with performance, in these situations dimensionality reduction methods can assist selection (37, 38). Metrics that exhibit lower co-linearity with others in the proposed multi-layered validity model can be prioritized as containing more independent (less redundant) information. This is a desirable quality since it allows for clearer resolution when assessing an athlete's physical abilities if each metric represents a distinct quality (1).

### 4.2 Decision making criteria

Decision making objectives consider the extent to which a user can knowingly act on the information contained in the measurement (4, 39) (Table 2). A key feature of this is interpretability, which reflects the capacity to analyze and integrate physical performance measures into training and competition protocols (i.e., the practical utility of the measurement) (21). While decisions can be made in the absence of interpretation, they are more likely to lead to errors and unintended consequences. Test measurements should align with specific objectives, such as performance enhancement, talent identification, or injury prevention, and should inform the development of physical training programs. Therefore, the influence of specific decision making factors may change depending which aspect of the physical preparation plan is of interest. To strengthen the interpretability of these metrics, normalization within a team or comparative benchmarking against competitors or comparable sports can offer a contextual reference, resulting in improved utility of the data (1, 40). A performance metrics’ responsiveness reflects its accuracy in detecting meaningful changes in response to a defined stimulus (41, 42). In physical preparation for sport, this stimulus is often characterized by training interventions, but can also be influenced by variables such as fatigue, adaptations from other training activities, or from competition itself. The end-user must also consider the principle of diminishing returns in the context of training stimulus and adaptation. Diminishing returns refers to the observation that as an athlete receives greater exposure to a stimulus, the rate of adaptation decreases, and alternate training stimuli are required to achieve continual physical performance gains (43–45). Therefore, as an athlete's training progresses, the relevance of a specific physical performance metric may decrease due to a plateau in potential adaptation in one physical domain, meanwhile another physical performance metric may gain prominence as it allows for a greater potential for physical performance improvement.

### 4.3 Organisational criteria

Organisational objectives (Table 3) reflect the feasibility of implementing a physical performance testing and analysis process within the sports organization. Feasibility assessments are commonly used in sport business, management, strategic planning, and marketing to determine the costs vs. benefits of business strategies, processes, projects, programs, and facilities (46). The costing considerations of selecting physical performance metrics pertains to the financial cost, time cost, and opportunity cost of undertaking the testing and analysis (21, 47). The inclusion of a feasibility evaluation of a physical performance metric provides practitioners and their organizations information on the cost vs. benefit of their testing battery. For example, a feasibility evaluation may allow practitioners to quantify the value of a performance test purpose (i.e., training intervention, talent identification, injury prevention), financial costs (i.e., staff wages, equipment, venue), time cost (i.e., set-up time, time athletes take to complete test, data analysis and interpretation, staff upskilling), physical risk vs. benefit of testing (i.e., injury, adverse training/performance affects), and identification of similar performance metrics assessed across multiple tests (21, 48).

### 4.4 Secondary criteria

Secondary criteria represent impacts that follow the implementation of a measure that are outside of the primary objective (4). This might include factors such as athlete motivation, feedback, teamwork, competition. Secondary objectives also consider whether the inclusion of a physical performance metric has a wider impact on the organization or sport beyond the performance department such as its ability to aid developmental pathways, or foster grassroots sport. Negative consequences can also be considered as a secondary objective. For example, a secondary objective assessment may identify potential scenarios where the physical performance metric to be used incorrectly, such as its use in the wrong sub populations. Errors leading to wrong decision-making can be costly for a sports organization as they result in wastage of resources, thus affecting the team's financial stability and may lead to budgetary constraints in other areas the sports organization. Secondary impacts may therefore range from internal financial implications within an organization, to those impacts that have a wider social or sports governance impact that is challenging to quantify.

## 5 Framework application

**Figure 3:**
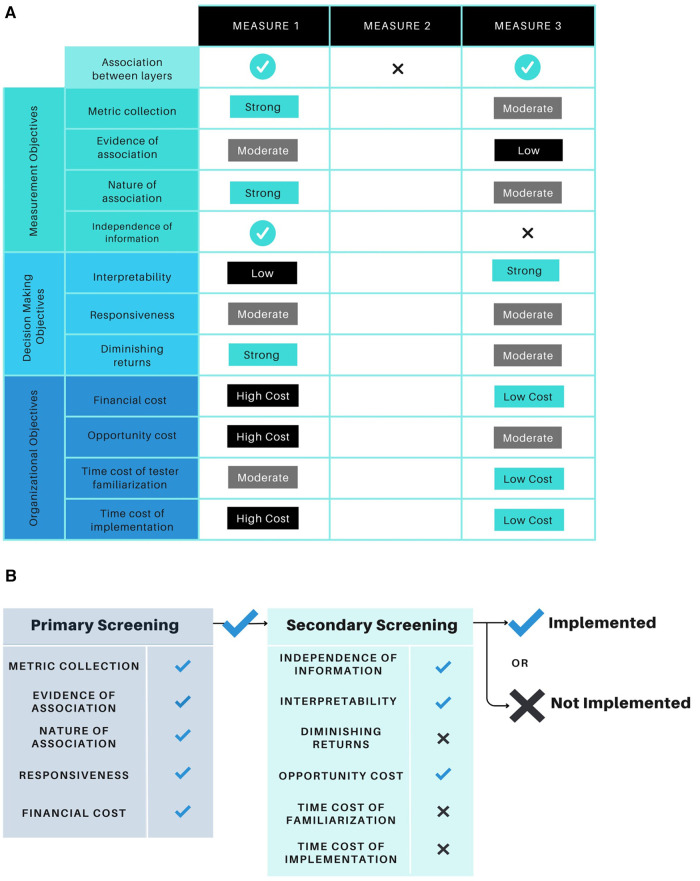
(**A**) A case example of how the framework can be applied via a checklist format. In this example, the tool commences with a single gatekeeper question, followed by each of the remaining criteria. The gatekeeper question states: “*Is there evidence of association between the measure and another “layer” in the validity model?*”. Without a positive response to this question, the remaining items and objectives are of little value. Adapted from Robertson et al. (18). (**B**) A case example of how the framework can be applied with items weighted differently based on their perceived importance. In this example, a potential measurement must past the primary round of screening before further consideration is given. The secondary round of screening may not require all selected items to be met prior to the measurement’s implementation into a testing battery. Adapted from Robertson et al. (18).

The proposed physical assessment framework has been developed to allow flexibility in its application across various sport contexts. At the broadest level, it can represent a checklist by which to compare multiple measurements that are being considered for implementation (18). In this example (Figure 3A), the tool commences with a single gatekeeper question, followed by each of the remaining criteria. The gatekeeper question states: “*Is there evidence of association between the measure and another “layer” in the validity model?*”. Without a positive answer to this question, the remaining items and objectives are of little value. This provides a broad, non-prescriptive model that can be used across a range of situations.

The framework may also be tailored to meet the specific needs of a physical preparation program, training environment, sports organization, or governing body. This can be done by weighting the items within each objective category more or less heavily than other items via multiple rounds of screening (18). In the example provided (Figure 3B) several items have been selected by an end-user as they may be deemed more important than others in their specific use case. In this example, a potential measurement must past the primary round of item and objective screening before further consideration is given. The secondary round of screening may not require all selected items to be met prior to the metric's implementation. As such, the secondary round of testing may serve to identify any limitations or constraints of a physical performance metric if the end-user decides to implement it within their training program or organization.

## 6 Conclusion

This article presented evidenced based processes to inform test measurement selection for the physical preparation of athletes. We described a method for building a multi-layered validity model, followed by a framework by which to evaluate the suitability of metrics that considers technical, decision-making, and organizational factors. The framework can be applied in several ways depending on the needs of the user, including a checklist design or a gatekeeper model. The systems presented here will assist in distilling the myriad of measurements available into those likely to have the greatest impact on performance.
